# Clinical Features of 50 Patients With Primary Adrenal Lymphoma

**DOI:** 10.3389/fendo.2020.00595

**Published:** 2020-09-24

**Authors:** Yan Wang, Yan Ren, Lifen Ma, Jian Li, Yuchun Zhu, Lianling Zhao, Haoming Tian, Tao Chen

**Affiliations:** ^1^Department of Endocrinology and Metabolism, Adrenal Center, West China Hospital of Sichuan University, Chengdu, China; ^2^Department of Endocrinology and Metabolism, People's Hospital of Deyang City, Deyang, China; ^3^Department of Endocrinology, Baoji Centre Hospital, Baoji, China; ^4^Department of Hematology, West China Hospital of Sichuan University, Chengdu, China; ^5^Department of Urology, West China Hospital of Sichuan University, Chengdu, China

**Keywords:** primary adrenal lymphoma, diffuse large B-cell lymphoma, high-density lipoprotein cholesterol, proto-oncogene, Epstein–Barr virus infection

## Abstract

**Background and Objective:** Primary adrenal lymphoma is a rare, progressive, easily misdiagnosed adrenal tumor with a poor prognosis. There are limited data on its clinical characteristics, and these have been derived from small sample studies. This study aimed to identify the clinical characteristics and prognosis of primary adrenal lymphoma.

**Methods:** This single-center study retrospectively analyzed data of 50 primary adrenal lymphoma patients treated between January 2008 and January 2018. Demographic information, biochemical indexes, computed tomography images, pathological findings, treatment regimens, and prognostic factors were analyzed.

**Results:** The median age of onset was 60.3 years, and 30 (60.0%) of 50 patients were male. Abdominal pain was the most common symptom, followed by incidentaloma and B symptoms. On average, patients presented with elevated lactate dehydrogenase (348 IU/L, normal range 110–220 IU/L) and hydroxybutyrate dehydrogenase levels (287 IU/L, normal range 72–182 IU/L) and decreased high-density lipoprotein cholesterol levels (0.88 mmol/L, normal range > 0.9 mmol/L). Bilateral lesions in the adrenal glands were observed in 30 (60.0%) patients. Computed tomography showed that 42 (84%) patients had signs of infiltration. Diffuse large B-cell lymphoma was present in 44 (88%) patients. Immunohistochemistry revealed that 70.6% (12/17), 89.5% (17/19), 92.0% (23/25), and 68.8% (11/16) of patients were positive for *MYC, p53, BCL2*, and both *MYC* and *BCL2*, respectively. Combined chemotherapy was associated with a good prognosis.

**Conclusions:** Early diagnosis of primary adrenal lymphoma depends on a combination of biochemical examination, imaging studies, and pathological biopsy, and combined chemotherapy may lead to a better prognosis.

## Introduction

In clinical practice, adrenal masses are frequently incidental findings on imaging studies that are not specifically performed for adrenal diseases. A systematic review demonstrated that the estimated risk for malignancies was 0.2% in all incidentalomas (1). Computed tomography (CT) is helpful for the diagnosis of benign and malignant tumors. However, there are limited data (only two studies involving 102 true incidentalomas) indicating that a CT density of >10 HU has high sensitivity for the detection of adrenal malignancies, This indicate that adrenal masses with a density of ≤ 10 HU are unlikely to be malignant (2). Among the diverse malignant causes of incidentalomas, a primary adrenal lymphoma (PAL) is rare causative tumor (2). In 2013, a systematic review indicated that <200 PAL cases have been reported in the English literature (3). Despite the rarity of this disease, PAL induces considerable clinical concern. First, despite the absence of benign characteristics, a PAL on CT and/or magnetic resonance imaging (MRI) may lead to misdiagnosis in the initial evaluation (4); furthermore in some cases, PAL is difficult to distinguish from adrenocortical carcinoma (ACC) (5) and pheochromocytoma (6). Second, PAL is a progressive disease with a poor prognosis, and some patients die before effective treatment can be administered (3). Therefore, early diagnosis is vitally important. Third, PAL belongs to non-Hodgkin lymphoma (NHL), While, diffuse large B-cell lymphoma (DLBCL) is the most common type, the pathophysiology of PAL has not been fully elucidated. Fourth, whether there is a strong association between NHL and decreased HDL-C is inconsistent in previous research (7, 8). Decreased average HDL-C levels and increased monocytes counts have been previously described in patients with non-Hodgkin lymphoma (9) and lymphoblastic leukemia (10). The underlying mechanism may be related to cytokines, such as interleukin-10, which are excessively secreted from lymphoma cells (11, 12). Inversely, a clear or strong association between these lipid traits and the most common NHL subtypes including DLBCL was not found, using the data of genome-wide association study from the Inter Lymph Consortium Mendelian randomization (MR) analysis (8). To date, the majority of published studies on PAL have been constrained by small sample size and insufficient clinical information. So far, this is the largest cohort of 50 patients with pathologically diagnosed PAL. Patients were evaluated retrospectively, to investigate the clinical characteristics and influence of treatment on prognosis over the course of a 2-year follow up period.

## Methods

From January 2008 to January 2018, 50 Chinese patients were pathologically diagnosed with PAL at the West China Hospital of Sichuan University in accordance with criteria that were suggested by a previous study. These diagnostic criteria included the following: a histologically proven lymphoma, no prior history of lymphoma, and dominant adrenal lesions in the presence of other organs and/or lymph nodes metastases (3). Histological classification of the lymphoma was based on the 2017 World Health Organization criteria (13). The positivity of molecular markers on immune-histochemical staining was attributed by the percentage of positive cells as follows: CD10 and BCL6 >30%, MYC >40% and BCL2 >50%. DLBCL was further classified into germinal center B-cell (GCB) and non-GCB subtypes by the Hans algorithm bases on the positivity of three markers (CD10, MUM1/IRF4, and BCL6) (14). The GCB subtype was characterized by CD10^+^/MUM1^−^, CD10^+^/MUM1^+^, or CD10^−^/MUM1^−^/BCL6^+^. The non-GCB subtype was characterized by CD10^−^/BCL6^−^, and CD10^−^/MUM1^+^/BCL6^+^ cases (14, 15). The presence of Epstein-Barr virus (EBV) RNA was examined by *in situ* hybridization (ISH) in lymphoma biopsy specimen. Moreover, fluorescence *in situ* hybridization (FISH) was performed to examine gene translocations (e.g., *MYC, BCL2*, and *BCL6*) (9).

Demographics information, biochemical indices, baseline cortisol, CT findings, pathological findings, treatment regimens, and prognosis, were collected for each patient. Adrenal insufficiency was defined as an early-morning (8–10 A.M.) serum cortisol <140 nmol/L and a plasma adrenocorticotropic hormone (ACTH) level 2-fold greater than the upper limit of the normal range (16). ACTH was determined using the electrochemiluminescence technique with the normal reference range being 7.2–63.3 pg/ml. Lipid profile was collected with the normal reference range being>0.9 mmol/L for HDL-C and 0.29–1.83 mmol/L for triglyceride (TG). The Ann Arbor classification was used for staging of PAL, based on the findings of the positron emission tomography–CT (PET-CT) or CT. This study was approved by the Ethical Committee of West China Hospital of Sichuan University (approval no. 2017-378) and adhered to the principles evinced in the Declaration of Helsinki and its later amendments.

Both MS Excel 2007 and R statistical software were used for the statistical analyses. Continuous data with normal and skewed distributions were described as the mean ± standard deviation (SD) and median (interquartile range), respectively, and were compared with an independent Student's *t*-test. Dichotomous data were compared using the chi-square test and Fisher's exact tests, and the results were described as percentages. The levels of high-density lipoprotein cholesterol (HDL-C), monocytes, lactate dehydrogenase (LDH), and hydroxybutyrate dehydrogenase (HBDH) were compared with the reference ranges of the general population from the same area. Cox proportional hazard models were used to evaluate the risk factors that contributed to the prognosis. A Kaplan–Meier approach was used to calculate overall survival. *P* < 0.05 was considered statistically indicative of statistical significance.

## Results

### General Clinical Characteristics

In this study population, 30 (60.0%) of the 50 patients were male. The median age at PAL onset was 60.3 years (range 21–84 years). Bilateral involvement was observed in 30 (60%) patients. With regard to comorbidities, six patients had hepatitis B infection, three patients each had tuberculosis and type 2 diabetes, two had gastric ulcers, two had lymphomas in other parts of body, one had small cell lung cancer, one had uveitis, one had thrombotic thrombocytopenic purpura, one had hemolytic anemia, one had rheumatoid-arthritis, and one had a human immunodeficiency virus (HIV) infection. None of the patients had a history of cancer and autoimmune adrenalitis.

**Figure 1:**
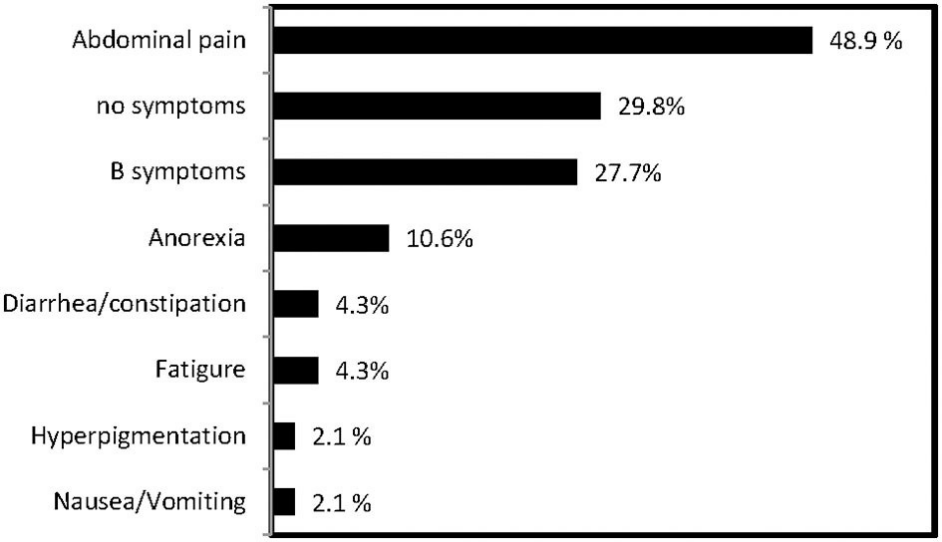
Frequency of clinical presentations of PAL in this series.

Except for three patients without data, 47 patients had a clear presentation of the characteristic clinical features of PAL. Abdominal pain was the most common symptom in 23 (48.9%) patients, followed by incidentaloma in 14 (29.8%) patients, B symptoms in 13 (27.7%, fever, night sweats, and weight loss), gastrointestinal symptoms in 7 (17.0%), fatigue in 2 (4.3%), and hyperpigmentation in 2 (2.1%) (Figure 1). In addition, hepatosplenomegaly was found in 4 of 37 patients (10.8%). Elevated LDH (30 of 37; 81.1%) and HBDH (28 of 37; 75.7%) levels were observed. The blood lipid profiles of patients were characterized by decreased HDL-C levels and elevated TG levels, whereas the total cholesterol and low-density lipoprotein cholesterol (LDL-C) levels were within the normal range. Patients' total white blood cell counts were within the normal range. An evaluation of the adrenal function in the 35 patients with available data showed that only 4 (11.4%) patients had baseline cortisol (17.64, 120.4, 79.24, and 94.86 nmol/L, respectively), and ACTH levels (216.2, 391.0, 219.9, and 325 pmol/L, respectively), that were suggestive of adrenal insufficiency, including 3 (8.5%) patients with bilateral adrenal involvement and 1 (2.9%) patient with unilateral adrenal involvement. All patients were treated with prednisone (5–10 mg daily). In 8 (38.1%) patients, EBV-RNA was detected in the serum (Table 1).

**Table 1:** Clinical characteristics of PAL patients.

	**Total**	**Reference**
*n*.	50	
Age(years)	60.3 ± 14.1	–
Male (n)	30 (60%)	–
Unilateral/bilateral involvement	22/28	–
BMI (kg/m^2^)^a^	22.2 ± 3.2	–
Malignances(*n*, %)	1 (2.0%)	–
Autoimmune diseases(*n*, %)	5 (8.0%)	–
Infectious disease (*n*, %)	10 (20%)	–
TG (mmol/L)^b^	1.49 ± 0.69	0.29–1.83
HDL-C (mmol/L)^b^	0.88 ± 0.38	>0.9
UA (μmol/L)^b^	329.2 ± 296.9	240.0–490.0
LDH (IU/L)^b^	571.2 ± 358.0	110–220
LDH %(*n*, %)^b^^,^ ^d^	30 (81.1%)	–
HBDH (IU/L) ^b^	444.5 ± 289.0	72–182
HBDH %(*n*, %) ^b^^,^ ^d^	28 (75.5%)	–
WBC ^b^	5.3 ± 2.2	3.5–9.5 × 10^9^/L
Neutrophils %	62.8 ± 14.9	40–75.0%
Lymphocytes %	21.1 ± 9.41	20–50.0%
Monocytes %	8.7 ± 2.9	3–10.0%
Adrenal insufficiency (*n*)^c^	4 (11.4%)	–
Bilateral adrenal involvement	3 (8.5%)	
Unilateral adrenal involvement	1 (2.9%)	
Serum Epstein-Barr virus-encoded small	8 (38.1%)	
Bilateral lesions (*n*, %)	30 (60%)	–
**Tumor Size (cm)**
Left (range)	6.5 (1.7, 12.1)	–
Right (range)	6.7(1.4, 16.8)	–

*Adrenal insufficiency was defined as an early morning (8–10 A.M.) serum cortisol level <140 nmol/L and a plasma adrenocorticotropic hormone (ACTH) level that was 2-fold greater than the upper limit of the normal range (17), because a corticotropin stimulation test is not feasible in the western area of China. The cortisol level of four patients with adrenocortical insufficiency were 94.86, 79.24, 120.40, 17.64 nmol/L, and corresponding ACTH level were 325, 219.9, 391, 216.2 pmol/L, respectively*.

a *Twenty-five patients with data regarding BMI*;

b *37 patients with data regarding TG, HDL-C, UA, LDH, HBDH level, and WBC count*;

c *34 patients with data regarding ACTH and early-morning cortisol level*;

d *(n) represented the number of patents whose LDH or HBDH level were above the upper limits of the reference range*.

*BMI: body mass index; TG triglyceride; UA uric acid; LDH: lactate dehydrogenase; HBDH: hydroxybutyrate dehydrogenase; HDL-C: high-density lipoprotein cholesterol*.

*6 HBV, 3 TB and diabetes respectively, 2 gastric ulcers, 2 lymphomas in other parts, 1 small cell lung cancer, 1 uveitis, 1 thrombotic thrombocytopenic, 1 hemolytic anemia and 1 rheumatoid arthritis. 1 HIV, and no patients with tumor history and autoimmune adrenalitis*.

### Radiography Findings

**Figure 2:**
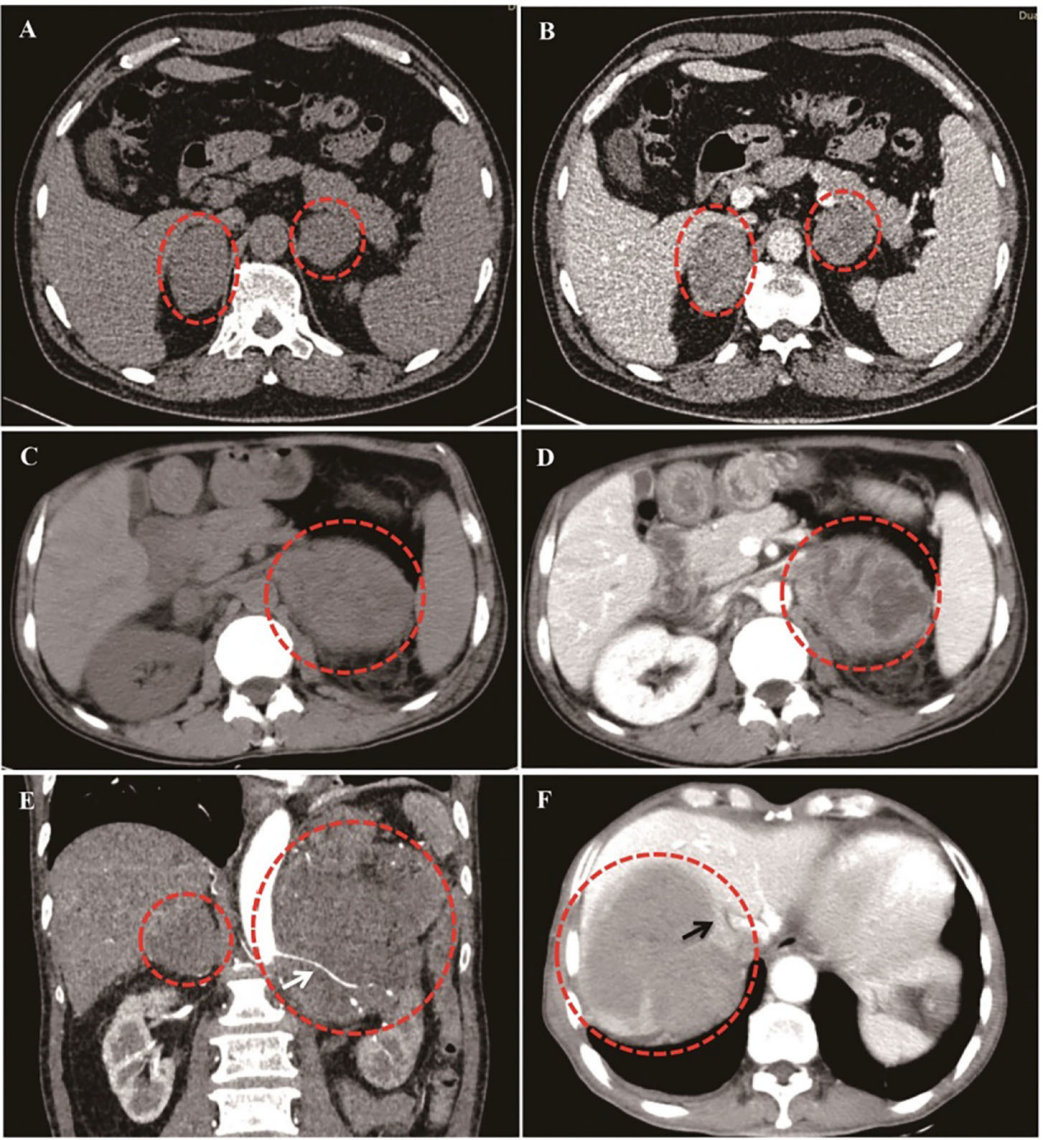
Different CT imaging features of PAL patients. **(A,B)**: Bilateral PAL with oval/round shape, well-defined edge, moderately enhanced in parenchyma phase, and homogeneous enhancement patterns. **(C,D)**: Bilateral PAL with liquidation/necrosis in the left tumor mass, which was obvious after contrast by iodamide. **(E)**: Lymphoma cells surrounded the renal artery without invasion (White arrow). **(F)**: Right lymphoma mass invaded inferior vena cava (Black arrow).

As shown in Figure 2, PAL patients demonstrated versatile CT findings with regard to tumor shape, edge, enhancement patterns, liquidation/necrosis, and local infiltration. Unenhanced CT findings were available for all patients and enhanced CT findings were available for 33 (66%) patients. Briefly, the adrenal glands were unilaterally and bilaterally affected in 20 (40.0%) and 30 (60%) patients, respectively. The median diameter of the lesions was 6.5 ± 2.7 cm (1.7–12.1 cm) and 6.6 ± 3.6 cm (1.4–16.8 cm) on the left and right sides, respectively. The edges of 26 lesions were well-defined, whereas 21 lesions had ill-defined edges. The median non-contrast CT density was 31.8 (25.8, 44.4HU). None of the lesions had a measurement value of <10 HU. On contrast imaging with iopamidol, moderate enhancement in the arterial and the parenchyma phases were observed bilaterally, and enhancement patterns were heterogeneous in 42.2% of patients. A total of 76.2% of patients presented with signs of infiltration of local organs or tissues (Table 2). Only two patients underwent MRI in our cohort, 1 case was characterized by an irregular mass with ambiguous boundary, measuring 5.3 × 4.1 × 3.9 cm^3^, around the left kidney with uneven low signal in the T1-weighted image, slightly high signal in the T2-weighted image, and obvious inhomogeneous enhancement on the enhanced scan. The MRI data of the other case detail was not available because the patient was being examined in another hospital. Additionally, PET/CT were examined in three patients. The results showed that the glucose metabolism in the adrenal was obviously increased. Additionally, one case was accompanied by the increased metabolism of glucose involving the abdomen and neighboring lymph nodes and liver. Seventeen patients underwent head CT or MRI screening, and only one patient had lymphoma invasion in the central nervous system.

**Table 2:** Extra-adrenal infiltrations.

**Affected organ/tissues**	***n* (%)**
Kidney vasculatures	19 (45.2)
Lymph nodes	16 (38.1)
Kidney	15 (35.7)
Inferior vena cava	10 (23.8)
Liver	7 (16.7)
Pancreas	6 (14.3)
Spleen	4 (9.5)
Osteomedullary invasion	4/25 (8.0)

*Forty-two patients were found to have extra-adrenal infiltration*.

### Pathological Features

A total of 36 patients were pathogenically diagnosed based on CT/ultrasound-guided needle core biopsy findings, and 14 patients were diagnosed after surgical resection. Of patients diagnosed with PAL, 44 (88%) patients were diagnosed with DLBCL, 4 (8.0%) with extra-nodal natural killer (NK)/T-cell lymphoma (nasal type), and 2 (4.0%) with peripheral T-cell lymphoma (PTCL). Of the 44 patients with DLBCL, 7 were further classified to have the GCB subtype, and 37 were classified into the non-GCB subtype. The seven patients with the GCB subtype showed the following profiles: two patients, CD10^+^/MUM1^−^, two patients, CD10^+^/MUM1^+^; and three patients, CD10^−^/MUM1^−^/BCL6^+^, The 37 patients with non-GCB subtypes showed the following profiles: four patients, CD10^−^/BCL6^−^ and 33 patients, CD10^−^/MUM1^+^/BCL6^+^. In Supplemental Figure 1, we present the pathological findings of a 59-year-old woman who was diagnosed with DLBCL. Immunohistochemical analysis showed that the tumor cells were positive for CD20, BCL2, BCL6, and MUM-1 and were negative for CD5 and CD10.

All patients a except for two showed strong positivity for Ki-67 (most >50%). Immunohistochemical analysis revealed that 70.6% (12/17) of patients were positive for *MYC*, 89.5% (17/19) for *p53*, 92.0% (23/25) for *BCL2*, and 68.8% (11/16) for both *MYC* and *BCL2*. EBV-RNA screening was performed by ISH in 25 patients, and four patients had positive results. Gene rearrangements were screened in 14 patients, 78.6% (11/14) of whom exhibited *IGH/IGK* rearrangement. FISH was further performed in five patients, three of whom carried the *BLC6* translocation and 1 had a *MYC* translocation (Supplemental Figure 2). Moreover, two of the five patients with T-cell lymphoma had TCRγ gene rearrangement, and both patients tested positive for EBV-RNA in lymphoma cells. Twenty-five patients underwent bone marrow (BM) biopsy, and 8.0% (*n* = 4) were found to have BM involvement.

### Factors Associated With the Prognosis

Of the 44 patients with available data, 14 patients had undergone surgical resection, and four subsequently accepted chemotherapy after surgical resection; A further 16 patients underwent chemotherapy after diagnosis based on CT/ultrasound-guided biopsy findings. Among the 20 patients who underwent chemotherapy, 15 had complete remission, four had partial remission, and one had one no remission. Fourteen patients only used glucocorticoid agents and/or supportive treatment due to poor clinical conditions at the time of diagnosis (Table 3).

**Table 3:** Types and schedules of chemotherapy and patient responses.

**Chemotherapy type**	Chemotherapy regimen (^*^)	**Case (*n*)**	**Response**
CHOP	1^*^	1	PR
	6^*^	2	2CR
	8^*^	3	3CR
	Unknown	3	1CR+1PR+1NR
R-CHOP	6^*^	2	2CR
	8^*^	1	CR
	Unknown	2	CR
CHOP+ R-CHOP+Gemox	CHOP × 2^*^+R-CHOP × 1^*^+Gemox × 6^*^	1	CR
R+V+P	1^*^	1	PR
Gemox	4^*^	1	CR
R-CHOP+ICE	R-CHOP × 8^*^+ICE × 4^*^	1	CR
GLIDE	4^*^	1	CR
P	2^*^	1	PR

*CHOP, Cyclophosphamide+ Doxorubicin+ Vincristine+ Prednisolone; R, Rituximab; V, Vincristine; P, Prednisolone; ICE, Ifosfamide+ Cisplatin+ Etoposide; GLIDE, Gemcitabino+ L-asparaginase+ Ifosfamide+ Dexamethasone+ Etoposide; Gemox, Gemcitabino+Oxaliplatin; CR, complete remission, PR, partial remission, NR, no remission*;

* *, chemotherapy regimen*.

The median follow-up duration was 22.45 months in 35 patients for whom data were available. The 1-year and 2-year overall survival rates were 79.5 and 72.5%, respectively, during follow-up. In this cohort, four patients died in the hospital without undergoing chemotherapy, and three patients died within 3 months to 2 years due to causes such as multi-organs failure (MOF) and severe infection.

Cox univariate proportional hazard models were employed to investigate the association of risk factors with the prognosis of PAL. The 2-year overall survival rate was 84.2% in patients who underwent chemotherapy, compared to 41.7% in patients who did not undergo chemotherapy (i.e., surgery alone and supportive treatment). The results showed that chemotherapy alone was associated with a lower risk of death (*P* = 0.029); and the mortality risk was 78.9% lower in patients who underwent chemotherapy compared to those who did not. The LDH, TG, and HDL-C levels, adrenal insufficiency, and international prognostic index scores were not associated with prognosis. Compared with the patients who were treated with chemotherapy and/or surgery to improve survival, the patients who only received palliative care had higher LDH and HBDH levels. In addition, baseline patient characteristics, including age, sex, HDL-C levels, pathologic type, and adrenal involvement, showed no differences between these two groups (Supplemental Table 1).

**Figure 3:**
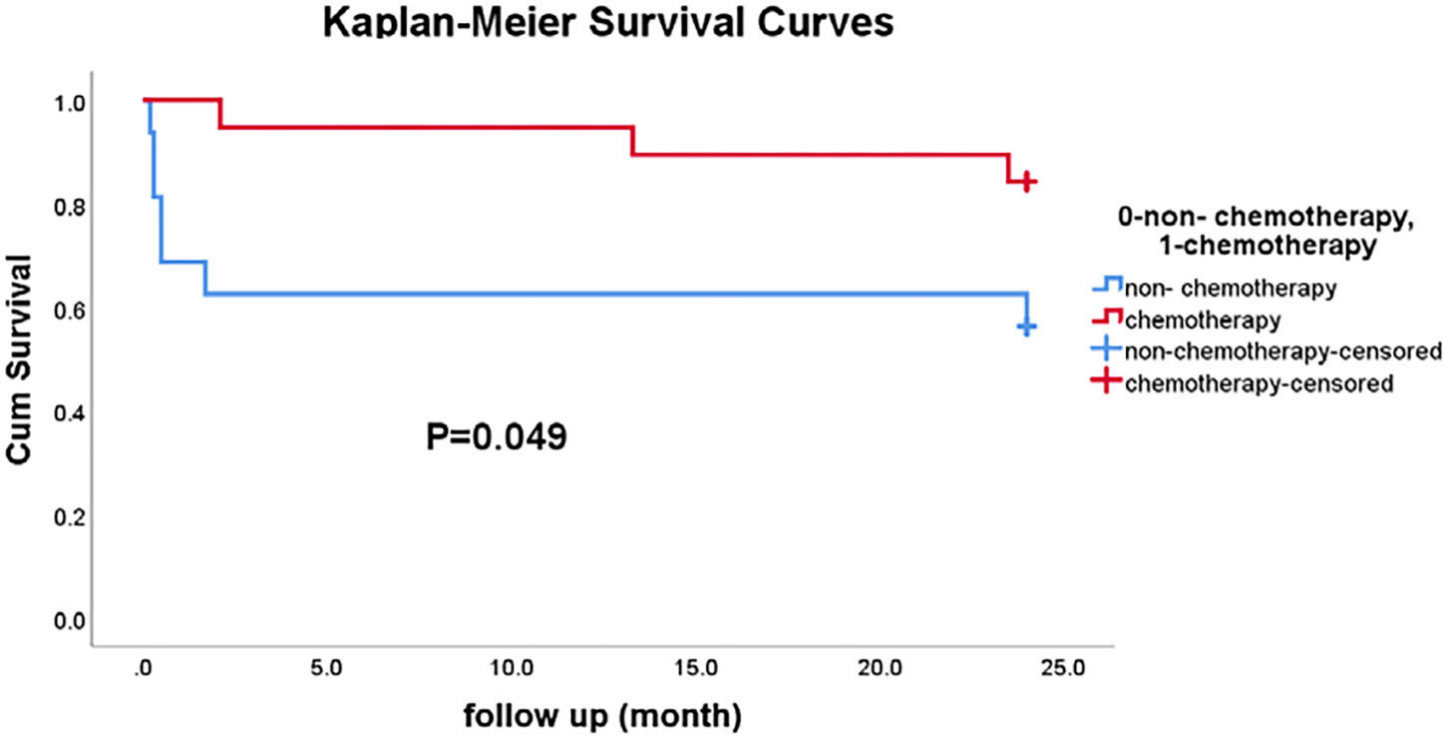
The comparison of 2-year survival rates between the chemotherapy group and the non-chemotherapy group.

The Kaplan–Meier approach was used to examine the difference in the overall survival between the chemotherapy group and non-chemotherapy group (Figure 3). There were 19 patients in the chemotherapy group (including four patients who received postoperative chemotherapy) and 16 patients in the non-chemotherapy group (including nine patients who received supportive treatment, 5 who underwent surgery, and 2 with an unknown treatment strategy). After 24-months follow-up, the survival rate was slightly significantly higher in the chemotherapy group than in the non-chemotherapy group (*P* = 0.049).

## Discussion

PAL is a rare but fatal disease, and understanding of its clinical manifestations, pathogenesis, and prognostic factors (3). Therefore, additional information is needed to better understand this disease. This study retrospectively analyzed the features of 50 PAL patients to evaluate their clinical presentations, laboratory results, imaging findings, pathologic details, potential mechanisms, and prognostic factors.

The median age at onset was ~60 years, and there were more male patients included than females (1.4 times more men than women), consistent with the results reported in several previous studies (5, 18). Notably, 29.8% of patients were asymptomatic, and PAL was detected during routine health checkups. This result was considerably different from that of a previous large case series, in which only a few patients (<1%) were identified incidentally (3). In another case series in Japan, two of five patients were asymptomatic (18). Furthermore, a few cases have been reported as adrenal incidentaloma without any symptoms (9, 19, 20). Strikingly, most of the asymptomatic patients had bilaterally disease, and three patients in this study and one patient in the previous study (18) developed a tumor mass with a maximum diameter of more than 8 cm. These results suggested the heterogeneity of PAL with regard to clinical manifestations and emphasized the differential diagnosis of adrenal incidentaloma, especially in those with bilateral lesions and larger sized tumors.

As expected, LDH and HBDH levels were increased in >80% of patients. In our series, the patients who only accepted palliative care had higher LDH and HBDH levels than those who were treated with chemotherapy and/or surgery to improve survival, whereas the patients who were treated with palliative care had worse conditions. Therefore, we speculate that high levels of LDH and HBDH are predictive factors for the poor prognosis of PAL. Moreover, this study demonstrated that lightly decreased average HDL-C levels and increased monocytes counts in PAL patients. Therefore, such changes in lipids profiles, white blood cell counts, and LDH/HBDH level seemed to be unique features of PAL in the setting of adrenal masses. However, our Cox multivariate proportional hazard model analysis also did not reveal an association between LDH, TG, and HDL-C levels and the prognosis of PAL.

It has been suggested that adrenal insufficiency in PAL is significantly related to older age, bilateral lesions, and hyper-segmentation (3). In our study, adrenal function was evaluated in 35 patients, and only 4 (11.4%) were found to have adrenal insufficiency: three patients had bilateral adrenal involvement, and one patient had unilateral adrenal involvement. Unilateral involvement may also be related to the patient's history of tuberculosis. The level of ACTH and baseline cortisol in patients with bilateral lymphoma who did not have adrenal insufficiency were 123.1 pmol/L (95% CI: 48.6, 197.6) and, 444.2 nmol/L (95% CI: 348.4, 540.1), respectively. In contrast, the incidence of adrenal insufficiency was largely less than those in previous studies, with a reported incidence of 61% (3). The unexpected commonness of adrenal insufficiency in PAL patients is likely due to biases related to publication and research methods (21). The data evaluated in this previous study were obtained from published literature, whereas our study used data from retrospective cases in a single medical center. Moreover, in our series, the incidence of bilateral involvement was 30 (60%), with an average age of 62 years vs. 75% bilateral involvement, with an average age of 60 years in previous research. Finally, only 54% of the cases in this previous study were from Asia, while all cases in our study were from Asian. This poses the question of whether the differences in the incidence of adrenal insufficiency were related to race; however, further observations and research are necessary to demonstrate this possible correlation.

The CT findings were similar to those in previous studies (3), including large tumor size, the incidence of slightly hypodense values in non-contrast CT scans, slight to moderate enhancement after the administration of iopamidol, a frequently observed heterogeneous appearance, and adjacent infiltration. Among these features, necrosis or cysts were observed in several patients and were misdiagnosed as pheochromocytomas or ganglioneuromas in the initial evaluation (data not shown), which is consistent with other reports (3, 22). Therefore, diagnosis of PAL at the initial evaluation based on the findings of traditional imaging modalities is challenging (23). Generally, the density of the adenomas is equal to or slightly lower than that of the normal adrenal gland tissue unless they are necrotic and/or cystic. The density is usually <10 HU in lipid-rich adenomas(70%) and between 10 and 30 HU in lipid- poor adenomas (30%). Our series show that the non-contrast CT density of PAL was 31.8 (25.8, 44.4) HU. However, it is still indistinguishable because of the similar appearance in adrenal lymphoma, pheochromocytoma, metastasis, and ACC. Heterogeneous enhancement, necrosis, and hemorrhage can be seen. Only a little sign can provide profitable alert for differential diagnosis. The enlargement of the retroperitoneal lymph nodes is common in adrenal lymphoma. The diversity in size was found in pheochromocytomas (17), It is usually larger than adenomas, but smaller than metastatic tumors, and the attenuation values are often similar to those of the muscle tissue, and are significantly higher than those of adrenal adenomas. Common primary malignancies and possible calcification were both found in metastases. Large masses (usually >6 cm) and invasion of the inferior vena cava is a common finding of ACC. Otherwise, MRI might be extra helpful for the differential diagnosis of PAL and pheochromocytoma or ganglioneuroma (23). Although T1 hypo-intense and T2 hyper-intense lesions were typically characteristic, a mild to moderate enhancement can be seen on the enhanced scan in adrenal lymphoma, isointense with ring-like or uneven enhancement after contrast administration may be found in adrenal metastases, and heterogeneous hyper-intensity was manifested in ACC. Regrettably, the one 1 case that had data available, was characterized by obvious uneven enhancement, rather than mild to moderate enhancement, which was inconsistent with the characteristics of previous literature reports (23). The value of MRI in these cases should be further evaluated in the future.

DLBCL was the commonest pathologic type (86%) in this cohort, consistent with the findings from other studies (3). Additionally, three patients had extra-nodal NK/T-cell lymphoma (nasal type), and three patients had PTCL. This distribution was different from that in previous reports, in which PTCL was more common, and only 3 NK/T-cell (nasal types) were identified among 187 cases of PAL (3). Among patients with DLBCL, seven were further classified as having the GCB subtype. A previous study suggested that those with the GCB subtype might have a better prognosis (24, 25). The longest complete remission period (56 months) was seen in a patient with GCB subtype, but the available data were not sufficient to draw a firm conclusion.

The etiology of PAL has not been fully elucidated. Immune dysfunction, EBV infection, and mutations in the *p53* and proto-oncogenes have been implicated (3, 21). In this cohort, few patients had a clear history of cancer, HIV infection, or autoimmune diseases, and no patients had a history of autoimmune adrenalitis, except for Hepatitis B virus infection (16.2%). Notably, this study revealed high positivity of *P53* (17/19), *BCL2* (23/25), and *MYC* (12/17). The phenomenon of *MYC* and *BCL2* co-expression was also frequently observed (11/16). Data on gene rearrangement are limited but suggested that the presence of the *IGH/IGK* rearrangement with *BCL2* or *MYC* expression might play a significant role in lymphoma genesis. With regarding EBV infection, only 4 of 25 patients exhibited the EBV-DNA in PAL tissues, this frequency was lower than that reported previously (45%) (26). Furthermore, a study showed that EBV infection seemed more active in T-cell lymphoma (27). All three patients with extra-nodal NK/T-cell lymphoma had EBV genomes in PAL tissues, TCRγ gene rearrangement might mediate EBV-induced T-cell transformation.

PAL is an aggressive lymphoma with strong Ki-67 expression in almost all patients, different from Castleman's disease with high interleukin-6 (IL-6) expression, heterotopic ossification, and benign micro-nodular hyperplasia appeared in proximity to the lymphatic tissue invading the adrenal (28). The reported survival rate approximately almost 20%, is poor at the 12-month follow-up (3). Due to the rarity of PAL, few studies have presented follow-up data.

Moreover, the 1-year and 2-year overall survival rates in the 35 patients with available follow-up data were 79.5 and 72.5%, respectively. This survival rate is higher than previously reported. Kim et al. (29) summarized 31 PAL cases and found that for patients with DLBCL treated with a median of six cycles of R-CHOP (cyclophosphamide, doxorubicin, vincristine, prednisone) chemotherapy, the 2-year overall and progression-free survival rates were 68 and 51%, respectively. With regard to the difference, early diagnosis could be one of the possible reasons; the initial indication of PAL was an incidentaloma during routine healthy examination in 29.8% of patients, which was significantly higher than that previously reported (1%) (30). Second, central nervous system involvement in PAL has a negative impact on long-term prognosis. In our series, only one patient had lymphoma invasion in the central nervous system.

Consistent with the findings of previous studies, chemotherapy, especially with the addition of rituximab, significantly improved outcomes (3, 29, 30). In early studies, the 1-year survival rate was nearly 20%, while in a recent series with more patients receiving rituximab add-on therapy, the 2-year survival rate was nearly 62% (5). In our analysis, the overall survival rates were 84.2 and 41.7% in patients who did and did not receive chemotherapy (surgery alone and supportive treatment), respectively. Meanwhile, the 2-year survival rate in the chemotherapy group was slightly higher than that in the non-chemotherapy group. The reasons for failure to undergo chemotherapy treatment included concerns about the effectiveness of the treatment, side effects, financial burdens, and severity of co-morbidities. Notably, two patients showed rapid deterioration and died in the hospital before chemotherapy could be administered, which further emphasizes the importance of early diagnosis of PAL according to clinical indications, e.g., adrenal imaging, alterations in LDH/HBDH levels, and changes in lipid profiles.

As a single center study, we collected the largest sample of PAL so far. Additionally we analyzed the clinical characteristics, pathological types, treatment, and prognosis. However, there are several limitations to the present study. A major limitation is its retrospective design, which may have led to missing or incorrect data; further, the assessment of adrenal function was insufficient. Another limitation is the incomplete follow-up data; only 15 patients completed a follow-up of more than 2 years. Furthermore, an inaccurate result could be deduced from the incomplete data. The third limitation is that more than half of the patients were diagnosed based on needle core biopsy findings, and in some cases insufficient tumor tissue was obtained for pathological analysis, especially for FISH. Otherwise, MRI was performed in only 2 cases. Hence, most of these deficiencies should be overcome when treating the adrenal incidentaloma again. The more complete evaluation including MRI was performed and clinical data were collected to accurately analyze the clinical characteristics of PAL and provide important reference for the choice of treatment strategy.

## Conclusions

In summary, this study systematically analyzed the clinical characteristics of 50 patients with PAL. Most patients developed PAL at the age of 60 years, with men making up the majority of the study cohort. Abdominal pain, incidentaloma, and B symptoms were the most common manifestations. Decreased HDL-C levels and lymphocyte counts and elevated LDH/HBDH levels demonstrated potential value for the differential diagnosis of PAL. DLBCL (non-GCB subtype) and extra-nodal NK/T-cell lymphoma (nasal type) were the most common pathological types. Moreover, tumor masses exhibited an aggressive growth pattern. Gene mutations (*P53, MYC*, and *BCL2*) and EBV infection played important roles in lymphoma genesis. Early diagnosis and combined chemotherapy were associated with a better prognosis.

## Data Availability Statement

The raw data supporting the conclusions of this article will be made available by the authors, without undue reservation.

## Ethics Statement

This study was approved by the Ethics Committee of West China Hospital of Sichuan University and adhered to the Declaration of Helsinki (No. 2017-378). The patients/participants provided their written informed consent to participate in this study. Written informed consent was obtained from the individual(s) for the publication of any potentially identifiable images or data included in this article.

## Author Contributions

TC and HT developed and designed the research. TC obtained funding, TC, YW, LM, and YR interpreted the data and drafted the manuscript. YZ and LZ contributed to data collection. All the authors revised and approved the final version of the manuscript.

## Conflict of Interest

The authors declare that the research was conducted in the absence of any commercial or financial relationships that could be construed as a potential conflict of interest.

## References

[1] Cawood TJ Hunt PJ O'Shea D Cole D Soule S. Recommended evaluation of adrenal incidentalomas is costly, has high falsepositive rates and confers a risk of fatal cancer that is similar to the risk of the adrenal lesion malignant; time for a rethink? Eur J Endocrinol. (2009) 161:513–27. 10.1530/EJE-09-0234 19439510

[2] Fassnacht M Arlt W Bancos I Dralle H Newell-Price J Sahdev A. Management of adrenal incidentalomas: european society of endocrinology clinical practice guideline in collaboration with the european network for the study of adrenal tumors. Eur J Endocrinol. (2016) 175:G1–34. 10.1530/EJE-16-0467 27390021

[3] Rashidi A Fisher SI. Primary adrenal lymphoma: a systematic review. Ann Hematol. (2013) 92:1583–93. 10.1007/s00277-013-1812-3 23771429

[4] Libe R Dall'Asta C Barbetta L Baccarelli A Beck-Peccoz P Ambrosi B. Long-term follow-up study of patients with adrenal incidentalomas. Eur J Endocrinol. (2002) 147:489–94. 10.1530/eje.0.1470489 12370111

[5] Laurent C Casasnovas O Martin L Chauchet A Ghesquieres H Aussedat G. Adrenal lymphoma: presentation, management and prognosis. QJM. (2017) 110:103–9. 10.1093/qjmed/hcw174 27795295

[6] Joseph FG Cook S Gowda D. Primary adrenal lymphoma with initial presentation concerning for bilateral adrenal pheochromocytomas. BMJ Case Rep. (2017) 2017:bcr2016219136. 10.1136/bcr-2017-220549 28830899 PMC5623247

[7] Lim U Gayles T Katki HA Stolzenberg-Solomon R Weinstein SJ Pietinen P. Serum high-density lipoprotein cholesterol and risk of non-hodgkin lymphoma. Cancer Res. (2007) 67:5569–74. 10.1158/0008-5472.CAN-07-0212 17522388

[8] Kleinstern G Camp NJ Berndt SI Birmann BM Nieters A Bracci PM. Lipid trait variants and the risk of non-hodgkin lymphoma subtypes: a mendelian randomization study. Cancer Epidemiol Biomarkers Prev. (2020) 29:1074–8. 10.1158/1055-9965.EPI-19-0803 32108027 PMC7196490

[9] Rizzo C Camilleri DJ Betts A Gatt A Fava S. Primary bilateral non-hodgkin's lymphoma of the adrenal gland presenting as incidental adrenal masses. Case Rep Med. (2015) 2015:620381. 10.1155/2015/620381 26681947 PMC4670633

[10] Halton JM Nazir DJ McQueen MJ Barr RD. Blood lipid profiles in children with acute lymphoblastic leukemia. Cancer. (1998) 83:379–84. 10.1002/(SICI)1097-0142(19980715)83:2<379::AID-CNCR24>3.0.CO;2-P 9669823

[11] Moraitis AG Freeman LA Shamburek RD Wesley R Wilson W Grant CM. Elevated interleukin-10: a new cause of dyslipidemia leading to severe HDL deficiency. J Clin Lipidol. (2015) 9:81–90. 10.1016/j.jacl.2014.09.014 25670364 PMC5513489

[12] Xiu B Lin Y Grote DM Ziesmer SC Gustafson MP Maas ML. IL-10 induces the development of immunosuppressive CD14(+)HLA-DR(low/-) monocytes in B-cell non-Hodgkin lymphoma. Blood Cancer J. (2015) 5:e328. 10.1038/bcj.2015.56 26230952 PMC4526782

[13] Polyatskin IL Artemyeva AS Krivolapov YA. Revised WHO classification of tumors of hematopoietic and lymphoid tissues, 2017 (4th Edition): lymphoid tumors. Arkh Patol. (2019) 81:59–65. 10.17116/patol20198103159 31317932

[14] Hans CP Weisenburger DD Greiner TC Gascoyne RD Delabie J Ott G. Confirmation of the molecular classification of diffuse large B-cell lymphoma by immunohistochemistry using a tissue microarray. Blood. (2004) 103:275–82. 10.1182/blood-2003-05-1545 14504078

[15] Choi WW Weisenburger DD Greiner TC Piris MA Banham AH Delabie J. A new immunostain algorithm classifies diffuse large B-cell lymphoma into molecular subtypes with high accuracy. Clin Cancer Res. (2009) 15:5494–502. 10.1158/1078-0432.CCR-09-0113 19706817 PMC7289055

[16] Bornstein SR Allolio B Arlt W Barthel A Don-Wauchope A Hammer GD. Diagnosis and treatment of primary adrenal insufficiency: an endocrine society clinical practice guideline. J Clin Endocrinol Metab. (2016) 101:364–89. 10.1210/jc.2015-1710 26760044 PMC4880116

[17] Tsirlin A Oo Y Sharma R Kansara A Gliwa A Banerji MA. Pheochromocytoma: a review. Maturitas. (2014) 77:229–38. 10.1016/j.maturitas.2013.12.009 24472290

[18] Harada K Kimura K Iwamuro M Terasaka T Hanayama Y Kondo E. The clinical and hormonal characteristics of primary adrenal lymphomas: the necessity of early detection of adrenal insufficiency. Intern Med. (2017) 56:2261–9. 10.2169/internalmedicine.8216-16 28794358 PMC5635296

[19] Bourne AE Bell SW Wayment RO Schwartz BF. Primary hodgkin lymphoma of the adrenal gland: a unique case presentation. Can J Urol. (2009) 16:4694–6. 19497184

[20] Ohkura Y Shindoh J Haruta S Kaji D Ota Y Fujii T. Primary adrenal lymphoma possibly associated with epstein-barr virus reactivation due to immunosuppression under methotrexate therapy. Medicine. (2015) 94:e1270. 10.1097/MD.0000000000001270 26252293 PMC4616607

[21] Mozos A Ye H Chuang WY Chu JS Huang WT Chen HK. Most primary adrenal lymphomas are diffuse large B-cell lymphomas with non-germinal center B-cell phenotype, BCL6 gene rearrangement and poor prognosis. Mod Pathol. (2009) 22:1210–7. 10.1038/modpathol.2009.87 19525926

[22] Falchook FS Allard JC. CT of primary adrenal lymphoma. J Comput Assist Tomogr. (1991) 15:1048–50. 10.1097/00004728-199111000-00030 1939757

[23] Fuqin W Junwei L Ruoxi Z Yonghua B Cailin L Bangguo L. CT and MRI of adrenal gland pathologies. Quant Imaging Med Surg. (2018) 8:853–75. 10.21037/qims.2018.09.13 30306064 PMC6177362

[24] Ide M Fukushima N Hisatomi T Tsuneyoshi N Tanaka M Yokoo M. Non-germinal cell phenotype and bcl-2 expression in primary adrenal diffuse large B-cell lymphoma. Leuk Lymphoma. (2007) 48:2244–6. 10.1080/10428190701636450 17926189

[25] Yoon SO Jeon YK Paik JH Kim WY Kim YA Kim JE. MYC translocation and an increased copy number predict poor prognosis in adult diffuse large B-cell lymphoma (DLBCL), especially in germinal centre-like B cell (GCB) type. Histopathology. (2008) 53:205–17. 10.1111/j.1365-2559.2008.03076.x 18752503

[26] Ohsawa M Tomita Y Hashimoto M Yasunaga Y Kanno H Aozasa K. Malignant lymphoma of the adrenal gland: its possible correlation with the Epstein-Barr virus. Mod Pathol. (1996) 9:534–43. 8733769

[27] Ali AS Al-Shraim M Al-Hakami AM Jones IM. Epstein- barr virus: clinical and epidemiological revisits and genetic basis of oncogenesis. Open Virol J. (2015) 9:7–28. 10.2174/1874357901509010007 26862355 PMC4740969

[28] Müssig K Horger M Wehrmann M. Adrenal castleman's disease. Ann Hematol. (2007) 86:63–5. 10.1007/s00277-006-0200-7 17039362

[29] Kim YR Kim JS Min YH Hyunyoon D Shin HJ Mun YC. Prognostic factors in primary diffuse large B-cell lymphoma of adrenal gland treated with rituximab-CHOP chemotherapy from the consortium for improving survival of lymphoma (CISL). J Hematol Oncol. (2012) 5:49. 10.1186/1756-8722-5-49 22889180 PMC3445827

[30] Ichikawa S Fukuhara N Inoue A Katsushima H Ohba R Katsuoka Y. Clinicopathological analysis of primary adrenal diffuse large B-cell lymphoma: effectiveness of rituximab-containing chemotherapy including central nervous system prophylaxis. Exp Hematol Oncol. (2013) 2:19. 10.1186/2162-3619-2-19 23915571 PMC3750298

