# Supplementary material for: Clinical Features of 50 Patients With Primary Adrenal Lymphoma
Source: Front Endocrinol (Lausanne). 2020 Sep 24;11:595. doi: 10.3389/fendo.2020.00595 (PMC7541938; doi:10.3389/fendo.2020.00595)
Supplement: Supplementary file 1 [file Table_1.DOCX]

# Supplemental figure 1 Immunostaining results of a PAL patient with the DLBCL type.


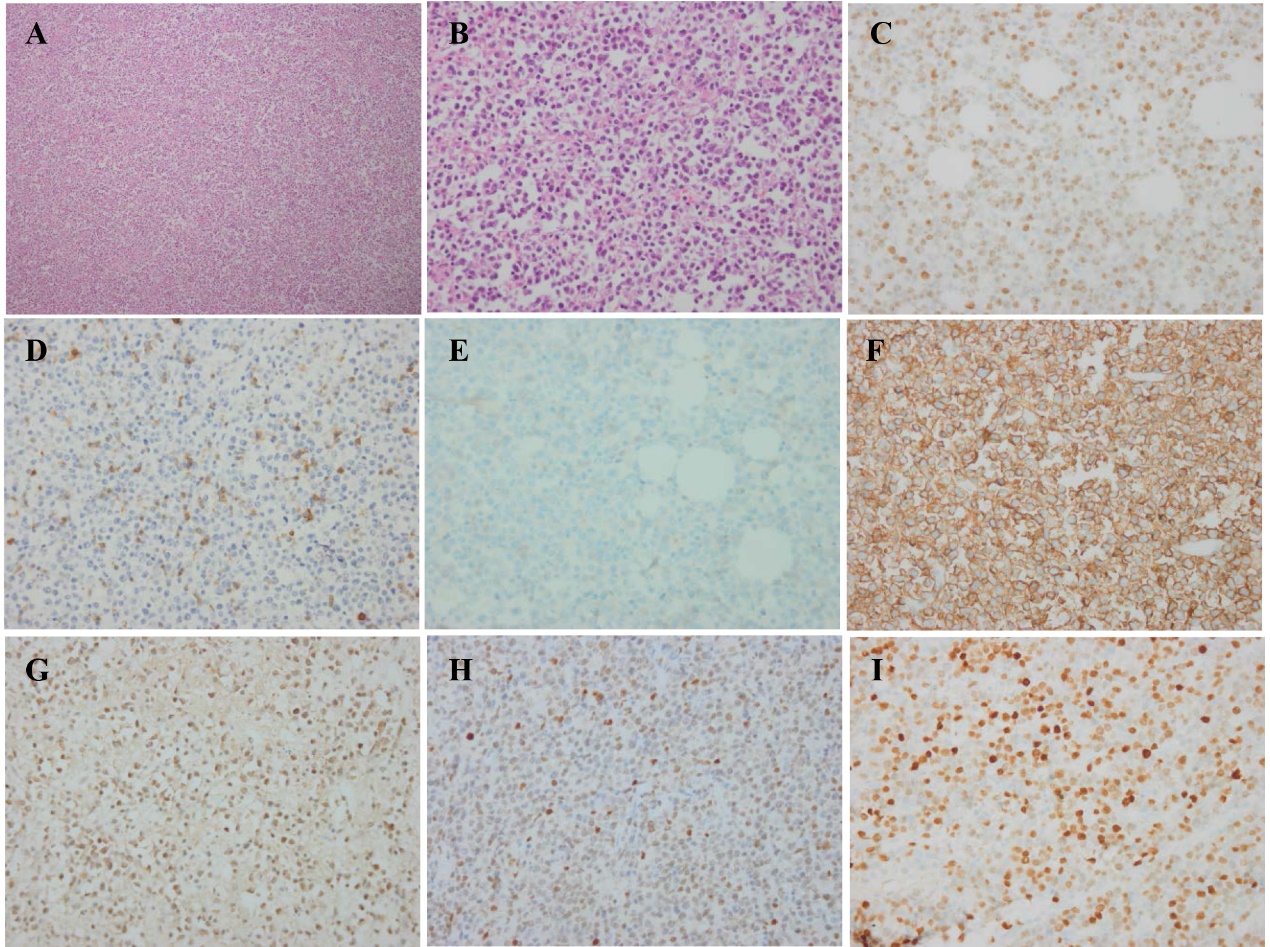


Pathological images from a 59-year-old female who was diagnosed as PAL(DLBCL). Section A and B were histopathologic photography (H&E staining, original magnification at 100×/400× respectively). Immunohistochemical analysis showed the tumor cells were positive for Ki-67 positive staining (>90%, section C), CD20 (section F), BCL2 (section G), BCL6 (section H) and MUM-1(I), while negative for CD5 (section D), and CD10 (section E).
