# Supplementary material for: Clinical Features of 50 Patients With Primary Adrenal Lymphoma
Source: Front Endocrinol (Lausanne). 2020 Sep 24;11:595. doi: 10.3389/fendo.2020.00595 (PMC7541938; doi:10.3389/fendo.2020.00595)
Supplement: Supplementary file 2 [file Table_2.DOCX]

# Supplemental figure 2 Fluorescence in situ hybridizations (FISH) for *BCL2*/*IGH* and *MYC*/*IGH* gene fusions.


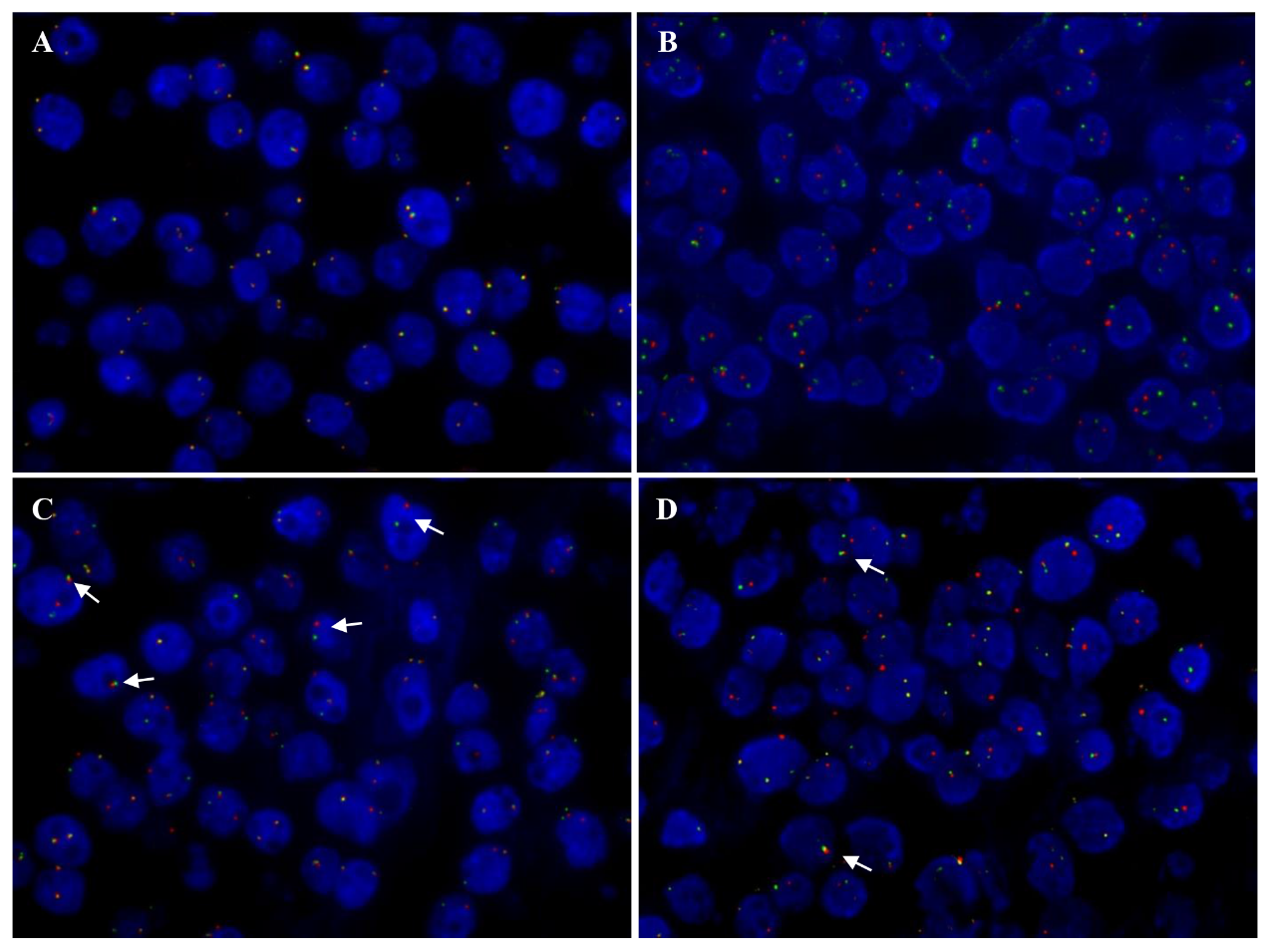


FISH results of the same patient as in Supplemental figure1. Section A and B showed negative results of *BCL2* gene broke down and *BCL2*/*IGH* gene fusion. Section C: Signals of *MYC* gene broke down (indicated by white arrow, Red = 5’ *MYC*, Green = 3’ *MYC*, Red/Green = intact); Section D: Fusion signals of MYC/*IGH* gene (indicated by white arrow, probe for IGH are Green, probe for MYC are Red).
