# Supplementary material for: Clinical Features of 50 Patients With Primary Adrenal Lymphoma
Source: Front Endocrinol (Lausanne). 2020 Sep 24;11:595. doi: 10.3389/fendo.2020.00595 (PMC7541938; doi:10.3389/fendo.2020.00595)
Supplement: Supplementary file 3 [file Table_4.DOCX]

Supplementary Table 1: Comparison of the baseline characteristics of patients who did or did not account for improved survival rates

|  | Group 1(n=28) | Group 2(n=22) | P Value |
| --- | --- | --- | --- |
| Age(y) | 60.1±12.7 | 61.1±16.3 | 0.22 |
| Gender(male/female) | 17/11 | 12/10 | 0.19 |
| Unilateral/bilateral adrenal involvement | 12/16 | 10/12 | 0.03* |
| LDH (IU/L) | 387.4±236.3 | 907.7±1508.2 | 0.023* |
| HBDH (IU/L) | 316.6±195.5 | 678.6±1054.5 | 0.034* |
| HDL-c(mmo/L) | 1.0±0.35 | 0.72±0.33 | 0.614 |
| Pathologic type  DLBCL  PTCL  Peripheral Cell Lymphoma | 25  1  2 | 19  2  1 | 0.78 |

Group 1: Surgery and/or chemotherapy were considered to improve survival rate. Group 2: Palliative care including CS and/or herbal were considered to not improve survival rate. Compared with Group 2, Group 1 had more bilateral adrenal involvement and lower LDH and HBDH levels. *: P＜0.05.
